# Novel statistical emulator construction for volcanic ash transport model Ash3d with physically motivated measures

**DOI:** 10.1098/rspa.2020.0161

**Published:** 2020-10-07

**Authors:** Qingyuan Yang, E. Bruce Pitman, Elaine Spiller, Marcus Bursik, Andrea Bevilacqua

**Affiliations:** 1 Earth Observatory of Singapore, Singapore, Republic of Singapore; 2 Asian School of the Environment, Nanyang Technological University, Singapore, Republic of Singapore; 3 Department of Materials Design and Innovation, University at Buffalo, Buffalo, NY, USA; 4 Institute for Computational and Data Science, University at Buffalo, Buffalo, NY, USA; 5 Department of Geology, University at Buffalo, Buffalo, NY, USA; 6 Department of Mathematical and Statistical Sciences, Marquette University, Milwaukee, WI, USA; 7 Istituto Nazionale di Geofisica e Vulcanologia, Sezione di Pisa, Pisa, Italy

**Keywords:** Ash3d, Gaussian Process emulator, volcanic ash fall deposit, machine learning, geophysical modelling, probabilistic hazard assessment

## Abstract

Statistical emulators are a key tool for rapidly producing probabilistic hazard analysis of geophysical processes. Given output data computed for a relatively small number of parameter inputs, an emulator interpolates the data, providing the expected value of the output at untried inputs and an estimate of error at that point. In this work, we propose to fit Gaussian Process emulators to the output from a volcanic ash transport model, Ash3d. Our goal is to predict the simulated volcanic ash thickness from Ash3d at a location of interest using the emulator. Our approach is motivated by two challenges to fitting emulators—characterizing the input wind field and interactions between that wind field and variable grain sizes. We resolve these challenges by using physical knowledge on tephra dispersal. We propose new physically motivated variables as inputs and use normalized output as the response for fitting the emulator. Subsetting based on the initial conditions is also critical in our emulator construction. Simulation studies characterize the accuracy and efficiency of our emulator construction and also reveal its current limitations. Our work represents the first emulator construction for volcanic ash transport models with considerations of the simulated physical process.

## 1. Introduction

### (a) Motivation

Statistical emulators are a powerful tool used in uncertainty analysis and statistical inversion. Given data, say from a moderate number of large numerical simulations that solve a system of differential equations, statistical emulators rapidly provide an approximation of the simulation output at untested input initial conditions, and give an estimate of the possible variability in that approximation [1]. In this way, once the emulator is well-trained, one could have a fast approximate estimate of the simulation output at an untested scenario. Further, the emulator offers a built-in measure of uncertainty for using the approximation in place of the computationally expensive simulation. Among many applications, emulators have been used in hazard quantification of geophysical processes (e.g.[2–14]).

In this paper we build a Gaussian Stochastic Process (GaSP) emulator as a surrogate model of the computer code Ash3d to examine the fallout of ash particles (tephra) consequent to a simulated volcanic eruption. Ash3d is a finite volume numerical model that simulates the transport and deposition of volcanic ash with different grain sizes released from a line source (i.e. an eruptive column) or a point source by solving the advection–diffusion equation in 3D. Ash3d requires many parameters as inputs, which include total eruption volume, column height, diffusion coefficient, tephra total grain size distribution and atmospheric conditions (see [15,16] for more details on Ash3d).

The presented emulator, once well-trained and under the condition of a relatively simple wind profile, can be used to predict simulated tephra thickness from Ash3d at locations of interest, and quantify the associated uncertainty in the estimate. Building emulators require a certain number of simulator (in this case, Ash3d) runs. Inputs and outputs from these runs are the training points for the emulator. In this work, the inputs refer to Eruption Source Parameters (ESPs) and the wind conditions and the output is the simulated tephra thickness at a location of interest.

The ultimate goal of constructing emulators is to have an efficient tool to aid in real hazard analysis (e.g. using the emulators to reconstruct a past eruption, or producing hazard maps for future eruptions). That said, such an analysis would warrant its own study. There are many sources of uncertainty, for example, physical processes and modelling with a paucity of data, that are not taken into account by Ash3d. Such uncertainties do not originate from the emulators, but would conflate with uncertainties introduced by using emulators. In this way, we would not be able to isolate, identify and evaluate the performance of the emulator. Instead the scope of this work is to construct emulators of Ash3D, and thoroughly understand their strength and limitations. Emulators-based hazard analysis for forecasting or reconstructing an eruption that combines models and data will be explored in future work.

As it is still unclear what the best way is to properly and effectively construct statistical emulators for volcanic ash transport models, we think that it is necessary to begin with relatively simple scenarios. More discussions on advantages and limitations of the presented emulator construction, rather than a declaration of a complete success and using it as a black box, are needed at the current stage. Careful examination and analysis of results from the constructed emulators are performed in this work. We present them here hoping that they could potentially help future studies inherit contributions from the present work, and build up and improve the current emulator construction accordingly, or avoid pitfalls noted in this work, and propose alternative, better solutions.

### (b) Previous studies

In using GaSP emulators we build on prior studies of geophysical flow models [17–23] and the construction of probabilistic hazard maps related to them [12,14,24–31]. Traditionally, a classic emulator construction involves a modest number of input variables (e.g. initial conditions and parameters in the governing equations), and yields a scalar output over those inputs. Extensions to vector-valued outputs have been developed [4], but the challenges and approaches to emulating Ash3d identified in this work will focus on the case of scalar output.

Sensitivity analysis, dimension reduction techniques and reduced order methods have been applied to reduce the number of input variables for the emulator [9,32–38]. In addition, robust methods for fitting emulators and careful consideration of the mean trend can improve the performance of an emulator [14,39–42].

Emulators or similar statistical strategies have been constructed or proposed for volcanic ash transport models in previous works [6,8,43–45]. The success of these studies comes from the use of novel techniques and ideas, and different ways to view and evaluate the data. Novel methodological developments will possibly also apply to other geophysical models.

In this work, we argue that to build up emulators for volcanic ash transport models, it is necessary to consider what is specific about the simulated process. The challenges and difficulties that are specific to building emulators for volcanic ash transport models need to be recognized, so that it is possible that they can be resolved.

Recent work of Shen *et al.* [46] implements dimensional analysis as a first step in the simulation process, prior to experimental design. Recall that dimensional analysis provides non-dimensional groups of parameters which determine the form of solutions to the governing system of equations [47]. Dimensional analysis helps to determine the effective parameters that best characterize a system and can reduce the number of parameter dimensions that must be explored by simulation. These ideas are analogous to the key concerns for the emulator construction of complicated simulators and motivate the present work.

### (c) Problem statement

The conventional way of training and constructing an emulator is by feeding inputs and outputs from simulator runs (as training data) to optimization packages that ‘fit’ the emulators. That is, they find the optimum parameters for the emulator based on the training data. In the conventional way, inputs and outputs of the simulator and emulator are identical, and no additional processes (e.g. subsetting and transformation of variables) are needed. In this work, two challenges described below prevent us from adopting the conventional way of emulator construction and they need to be addressed for properly constructing emulators for Ash3d. The two challenges will be addressed by using prior knowledge about the simulation process, which informs a transformation of input (e.g. summation or more complicated operation on certain input variables because there might be groupings of input variables that better capture variations in model output than individual variables do) and output variables and a subsetting of the training data points (i.e. subset the training data based on certain rules and train them seperately by subsets) before the parameter fitting.

#### (i) What (input) variables should be used to describe the ambient wind field for the emulator?

The wind field can be described by wind direction and speed, or equivalently, wind speeds along the longitude and latitude directions, at every elevation level. This is the wind profile format that can be used by Ash3d. However, it is impractical to use all wind field data as inputs to train the emulator. This is because using wind speeds and directions (or just wind speeds) at all elevations increases the number of inputs into the emulator (i.e. increasing the dimensionality of the input space), which is notoriously challenging for computationally expensive simulators, likely making the emulator infeasible to construct. Finding low dimensional inputs that adequately capture a spatial field of input data is an active area of research [48,49]. Specifically in this work, we need to find and use fewer and select forms of variables that are capable of capturing key and effective characteristics of wind profiles as inputs to train the emulator.

The most conventional way to proceed would be to parametrize the wind speed profile. For example, we tried to use a Gaussian profile to describe it. Without considering the change in wind direction, this only requires three variables, i.e. centre (mean) and standard deviation of the Gaussian profile and the maximum wind speed. This, however, creates another problem—the released tephra particles are only affected by a portion of the wind speed profile. Wind speed at high elevation does not affect tephra dispersal regardless of its value when the source height is low, but they would be greatly effective when tephra is released from a high elevation. The three variables used to define the wind speed profile may in fact contain pieces of information not affecting the output.

Whether the wind speed is fully effective or not depends on the tephra release height. Using the three variables (used to define the Gaussian wind profile) as inputs to train the emulator would have a negative impact on the performance of the emulator because their values do not always affect the output. This problem can be mitigated if an extremely large training dataset is available, but again, this is not possible given the high computational cost of numerical models. In this work instead we introduce the use of new, not trivial variables (detailed in the following) that can characterize the wind speed mixed together with the eruptive source parameters in an effective way.

#### (ii) How to account for the interaction between wind conditions and tephra particles with different grain sizes in training the emulator?

Depending on the grain size (which affects the terminal falling velocity of the grain), tephra particles react differently to the same wind profile. Therefore, the construction of the emulators is more complex under realistic polydisperse conditions. If we attempt to address the first challenge stated above (i.e. finding the appropriate variables to describe the ambient wind field), we need to make sure that the solution can be well incorporated into the fact that particles of different sizes are released from the source.

Total grain size distribution of released tephra is typically described by a lognormal distribution and characterized by median and standard deviation (as adopted in this work). It is possible that not all but only tephra particles with certain grain sizes are able to reach the location of interest. Hence values of the median and standard deviation of total grain size distribution could only partially affect the simulated tephra thickness. For tephra particles with a certain grain size, whether their amount would affect the simulated tephra thickness or not depends on factors such as wind conditions and altitude of release. Similar to the issue with the wind speed at high elevations, median and standard deviation of the total grain size distribution contain both effective (the ratio of certain grain sizes that would reach the location of interest) and non-effective (the ratio of certain grain sizes that cannot reach the location of interest) information. In this study, we seek for measures that are capable of accounting for the interaction between wind conditions and tephra particles of different grain sizes (and source height) in constructing the emulator.

### (d) Propositions

In this paper we advocate for applying the idea of dimensional analysis as part of the emulator construction process. We argue that there are groupings of input variables that better capture variations in model output than individual variables do. In such a case, these variable groups must be related to the physics of the simulated process. At the same time, we note that these variables might be heuristic: if an analytical solution or perfect variables to characterize the system exist, the emulator could be constructed in a conventional way (i.e. treating the simulator as a black box, just focusing on its inputs and output, and ignoring the simulated process in the emulator construction).

In this work we propose to construct the emulator by (i) scaling the output, and searching for the appropriate heuristic variables as inputs for the emulators, and (ii) subsetting the training data, and training them by subsets separately. These two actions disambiguate the many factors that influence ash fallout, providing a clearer process for emulation. From the viewpoint of machine learning, our idea can be phrased as finding the appropriate feature space and its subspace for the emulator (e.g.[50–53]), and this is done with the help of our prior knowledge about the process analysed.

Let us illustrate this idea by an example. Particles, even if they are identical in composition, fall out at different rates, depending on their size and the ambient wind. A larger particle released into a stronger wind could travel further than a smaller particle released into a weaker wind. However, when sampling in the particle size–wind speed parameter space (i.e. two sets of input initial conditions in Ash3d), the total distance travelled due to advection is determined by the interacting effects of the parameters (particle size, release height and wind speed). In the work reported herein, scaling particle fallout by accounting for particle size and the ambient wind velocity is key to calculating a practical emulator of tephra deposition.

### (e) Significance

The transport and deposition of volcanic ash pose threats to local community, infrastructure and aviation safety [43,54–62]. Probabilistic ash fall hazard prediction is a necessity for regions potentially exposed to volcanic activities [63–74]. Monte Carlo approaches to probabilistic ash fall hazard analysis require thousands of simulator runs. As such, emulation of the simulator is a more practical and efficient means of determining hazard and assessing uncertainty in the hazard analysis. Indeed, in the event that a volcano is about to erupt, hazard predictions need to be updated very rapidly, based on variable conditions such as wind speed and direction. Because of the efficiency of emulators, through an analysis of emulator construction the work here will enable fast and efficient probabilistic ash fall hazard prediction.

It is known that numerical volcanic ash transport models cannot simulate all physical processes (e.g. different dynamics near vent) taking place during an eruption, and that simplifications almost always exist in such models. Besides, in reconstructing a volcanic eruption, uncertainties arising from inaccurate knowledge of source and environmental parameters could affect the simulated results and the corresponding interpretations.

To better understand whether and how simplifications in numerical volcanic ash transport models would affect their ability to reconstruct an eruption (reproduce reality), and to take into account the uncertainties arising from inaccurate knowledge, a lot of numerical model runs are necessary. This is not always possible given the high computational cost of such models. Well-trained statistical emulators, because of their negligible computational cost, can thus be used as effective tools to improve our understanding of the performance and relationship between input and output (e.g. sensitivity analysis) of numerical volcanic ash transport models, and potentially enable efficient probabilistic inversion of volcanic eruptions.

Viewed from a technical perspective, our work marks the first attempt to construct emulators for volcanic ash transport models in a non-conventional manner.

### (f) Text organization

The fundamental idea we explore in this paper is to introduce measures to allow for an easier construction of the emulator based on our prior knowledge of the simulated process. We proceed by providing a brief summary of the GaSP emulator and the Ash3d solver. As part of the Ash3d discussion, we introduce a simplified advection–diffusion equation that can be solved analytically; this solution will play an important role in our emulator construction. Three new variables are introduced to characterize the wind conditions, and the wide spectrum of grain sizes are accommodated by training the emulator over subsets of inputs where the subsetting rule is based on the simplified analytical solution. We provide numerical tests of the emulator, not only to demonstrate its success but also to suggest further refinements of our approach. We list and analyse novelties, simplifications, sources of uncertainty and implications from our emulator construction in the discussion section.

## 2. Background

### (a) The GaSP emulator

An emulator estimates the output of a simulator for a set of inputs: initial conditions, parameter values, boundary conditions, etc., at which the simulator has not been run. The emulator can be regarded as an interpolator in spatial data analysis with the coordinates replaced by initial conditions, parameters and/or transformed variables based on them—generically ‘inputs’—required for the simulator. In this section, we give a brief introduction to the GaSP emulator. See Santner *et al.* [75] and Williams & Rasmussen [1] for more details on the theory and application of the GaSP emulators.

Output from the simulator evaluated at input ***x*** is denoted by **y**^*M*^(***x***). This output is viewed as the realization of a random process

2.1 $$y^{M}(x)=h(x)\beta +Z(x),$$

where ***h***(***x***) is a matrix of basis functions (typically low-order), and **β** is a vector of coefficients. Their product could give the overall trend of the data. Whether to define them or not (i.e. leave this term as constantly zero or not) and how to define them (their forms) depends on knowledge of the simulated process. For example, in constructing emulators for the geophysical flow model Titan2D, Spiller *et al.* [14] and Rutarindwa *et al.* [12] adopt the natural assumption that flow height at a location of interest is a monotonically increasing function of total flow volume (which is one of the initial conditions), and fit a linear mean in that direction. Based on this assumption, in their case, ***h***(***x***)**β** is the product of total flow volume and a coefficient plus a constant (the intercept of a one-variable linear function). *Z* is a zero-mean Gaussian process—that is, a stochastic process whose marginal distribution for every finite dimensional ***x*** is multi-variate Gaussian. *Z* is determined by its covariance, and we assume that $\text{Var}[Z(x)]=\sigma_{z}^{2}$ is a constant [37]. In this paper, we use the product power exponential as the correlation function. Specifically, if $x_{i}={\left(x_{i}^{1},x_{i}^{2},\ldots ,x_{i}^{k}\right)}^{T}$ is a vector in the input space of dimension *k*, the correlation function can be written as:

2.2 $$R_{ij}=C[z(x_{i}),z(x_{j})]=\prod_{k}\exp(-\gamma_{k}|x_{i}^{k}-x_{j}^{k}{|}^{\alpha_{k}}),$$

where the power {*α*_*k*_} characterize smoothness of the correlation function, and {*γ*_*k*_} are range parameters, denoting how rapidly the correlation decreases as the ***x***s move apart.

To construct the emulator, we select *N* points (***x***^*D*^ = {***x***_1_, …, ***x***_*N*_}) from an experimental design (the corresponding simulator output: **y**^*D*^), run the simulator for these input values and collect the outputs. Training of the emulator amounts to estimating the optimum values of **β**, *α*_*k*_, *γ*_*k*_ in equations (2.1) and (2.2) based on the training data.

The GaSP emulator estimates the output of the simulator for an untested point (x*). The estimated mean and variance, conditioned on the training data, can be written as:

2.3 $$\hat{y}(x^{*})=h(x^{*})\beta +r^{T}R^{-1}(y^{D}-h(x^{D})\beta ),$$

and

2.4 $$s^{2}(x^{*})=\sigma_{z}^{2}(1-r^{T}R^{-1}r+\frac{{\left(1-1^{T}R^{-1}r\right)}^{2}}{1^{T}R^{-1}1}),$$

where **r** = (*C*[(x*, ***x***_1_)], …, *C*[(x*, ***x***_*N*_)])^*T*^, and **R** is the correlation matrix whose (*i*, *j*)th element is given by *R*_*ij*_ in equation (2.2), and **1** is an *N*-dimensional column vector of ones. In this paper we use the R package ‘RobustGaSP’ [4,39,76] for training the emulator and making predictions.

### (b) The Ash3d simulator

Ash3d [16] is an Eulerian model that simulates the transport and deposition of tephra during explosive volcanic eruptions. It uses a robust, high-order, finite-volume method to solve the advection–diffusion equation in three dimensions [15]:

2.5 $$\frac{\partial q}{\partial t}+\nabla \cdot [(u+v)q]-\nabla \cdot (K\nabla q)=Q,$$

where *q* is the tephra concentration, **u** is 3-D wind vector, *v* is (terminal) tephra settling velocity, *K* is turbulent diffusivity and *Q* is a source term. It should be noted that the terminal velocity depends on the grain size as well as on atmospheric conditions, which can vary with elevation. Ash3d assumes that tephra is continuously released from a vertical line (or resembling the shape of a volcanic plume) or point source (depending on how the input is specified) with a constant mass flux rate over a prescribed period of time, and is transported in the atmosphere subject to wind advection, turbulent diffusion, and falling at the terminal velocity in the vertical direction.

To run Ash3d, the source term and turbulent diffusivity in equation (2.5) are defined by the user (see more details in [15,16]). By specifying the total volume and duration of the eruption and tephra density and grain size distribution, the total mass flux rate can be calculated. This value is then used as the mass flux rate for the source cell in Ash3d, if a point source is specified. Otherwise the vertical distribution of mass flux rate along the line source follows a uniform or Suzuki distribution [77]. Ash3d has been used by many scientists to model the transport and deposition of volcanic ash over a large area [70,78–81].

In this work we assume that volcanic ash is released from a point source. Ash3d provides several different methods to calculate the terminal velocity. Here the method of Wilson & Huang [82] is adopted for all simulations. Different formats of atmospheric condition data can be used as input for Ash3d. The one adopted in this work is a time-invariant wind profile, which specifies the temperature and pressure of the atmosphere and wind speed and direction at different elevation levels at one point. Although we could arbitrarily define values of the wind speed and direction at different elevations, we further assume a constant wind direction (northerly wind fixed in all elevation levels) for all simulations, with a speed that may vary with elevation in this work. This simplification is done so that we could implement our analysis with relatively fewer variables to consider, and to reduce the input dimensionality. As stated earlier, we need to know how to construct emulators in simplified scenarios before moving on to more complicated setups. Ash3d produces several output files, such as ash concentration field at user-specified times or the resultant tephra thickness distribution. This paper is concerned with tephra deposition, so examines the latter.

### (c) Tephra deposition with simplified assumptions: a semi-analytical solution

The solution to tephra thickness or mass per unit area distributions can be derived analytically under simplified assumptions. This approach has been widely used for studies on tephra fall deposits (e.g.[38,58,77,83–85]). Let us bin the grains into a discrete set of sizes *ϕ*(*j*), where *ϕ*(*j*) refers to grains with size (in millimetres):

2.6 $$\phi (j)=2^{\left(-j\right)}.$$

This is the Krumbein *ϕ* scale, which is adopted in Ash3d in this work, and *j* is an integer here. Assuming an instantaneous point source at an elevation *H*, and negligible turbulent diffusion and no wind in the vertical direction, the mass per unit area *m*(*χ*, *ψ*) of a tephra deposit at coordinates (*χ*, *ψ*) can be decomposed into a sum of terms representing the mass per unit area for particle size *ϕ*(*j*), $m(\chi ,\psi )=\sum_{j=\phi_{\text{min}}}^{\phi_{\text{max}}}m_{j}(\chi ,\psi )$. Here we use the unusual notations *χ* (east–west) and *ψ* (north–south) to denote coordinates in order to avoid using *x* and *y* repeatedly. Each *m*_*j*_(*χ*, *ψ*) is proportional to a two-dimensional isotropic Gaussian distribution [77], and can be written as:

2.7 $$\begin{array}{rl} & m_{j}(\chi ,\psi )=M_{j}f_{j}(\chi ,\psi )\\ \text{and}\; & f_{j}(\chi ,\psi )=\frac{1}{2\pi \sigma_{j}^{2}}\exp\left(-\frac{{\left(\chi -{\bar{\chi }}_{j}\right)}^{2}+{\left(\psi -{\bar{\psi }}_{j}\right)}^{2}}{2\sigma_{j}^{2}}\right), \end{array}\}$$

where *M*_*j*_ is the total mass of tephra with grain size *ϕ*(*j*), $\sigma_{j}^{2}=2KT_{0,j}$, and *T*_0,*j*_ is the total falling time from source height *H* to the ground for tephra particles with grain size *ϕ*(*j*).

$\left({\bar{\chi }}_{j},{\bar{\psi }}_{j})=(\chi_{s}+\sum_{i=1}^{n}\delta \chi_{i,j},\psi_{s}+\sum_{i=1}^{n}\delta \psi_{i,j}\right)$ denotes the coordinates of the plume centre when it reaches the ground, with (*χ*_*s*_, *ψ*_*s*_) being the source vent coordinates. $\sum_{i=1}^{n}\delta \chi_{i,j}$ and $\sum_{i=1}^{n}\delta \psi_{i,j}$ denote the total distance travelled by the plume centre in the *χ* and *ψ* directions, respectively, due to wind. Equation (2.7) implies that $\sum_{i=1}^{n}\delta \chi_{i,j}$ and $\sum_{i=1}^{n}\delta \psi_{i,j}$ are key to characterizing the effect of wind advection.

$\sum_{i=1}^{n}\delta \chi_{i,j}$ and $\sum_{i=1}^{n}\delta \psi_{i,j}$ are computed by separating the atmosphere into *n* horizontal layers of thickness Δ*H*_*i*_ with *i* = 1, 2, 3,.., *n*, and the settling time within each layer is calculated for particles with various grain sizes. The product of the settling time *t*_*i*,*j*_ and wind velocity within the *i*th elevation layer determines the advected distances *δχ*_*i*,*j*_ and *δψ*_*i*,*j*_. For example, *δψ*_*i*,*j*_ = (Δ*H*_*i*_/*v*_*i*,*j*_) *u*_*iψ*_ where *u*_*iψ*_ and *v*_*i*,*j*_ denote the wind speed in the *ψ* direction and terminal velocity of volcanic ash with grain size *ϕ*(*j*) in the *i*th horizontal layer, respectively.

Equation (2.7) suggests that (i) *m*_*j*_(*χ*, *ψ*) is proportional to the total mass of particles with grain size *ϕ*(*j*), and (ii) sums of advected distances, namely $\sum_{i=1}^{n}\delta \chi_{i,j}$ and $\sum_{i=1}^{n}\delta \psi_{i,j}$, play a key role in determining the value of *m*_*j*_(*χ*, *ψ*). These two features of equation (2.7) will guide our selection of variable groups that will be used.

### (d) The simplified case and Ash3d simulations

The semi-analytical solution described in the previous section is valid assuming instantaneous release of tephra particles from a point source and negligible turbulent diffusion and wind speed in the vertical direction. If the released particles all had the same grain size, the ash cloud would look like a flat disc that is spreading out in the *χ* and *ψ* directions (due to turbulent diffusion), falling at terminal velocity, and translating horizontally due to wind advection.

**Figure 1.**
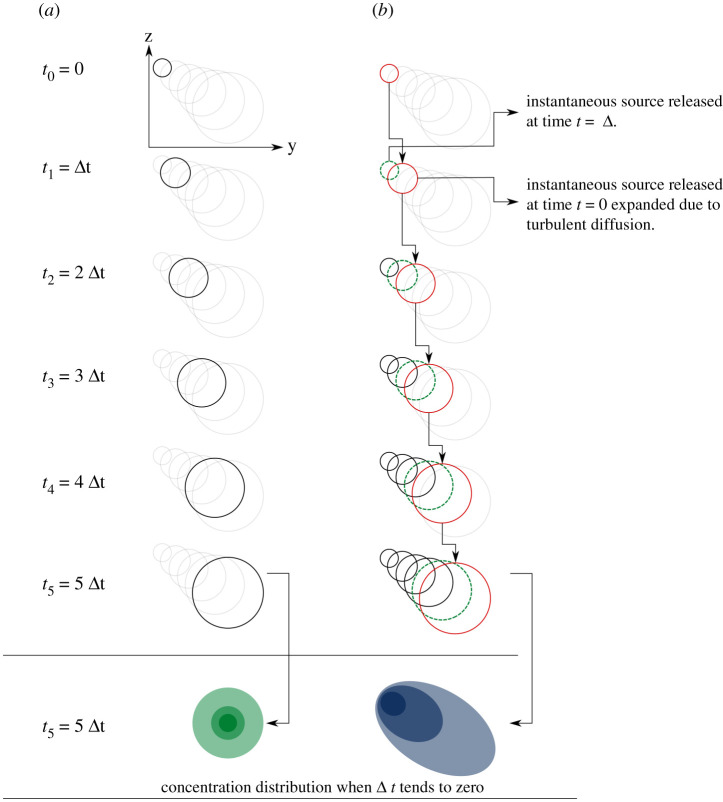
Schematic drawing highlighting the difference in plume shape (iso-concentration profile) between instantaneous (*a*) and continuous sources (*b*). In (*a*), the plume expands and transport is due to turbulent diffusion. In (*b*), the continuous source can be discretized to five instantaneous sources released at each moment for easier illustration. The shape of the plume (red circles) released at *t*_0_ is identical to the case with instantaneous source (*a*) at every moment. Similarly, the plume (green dashed line) released at *t*_1_ = Δ*t* at time *t*_1_, *t*_2_, …, *t*_5_ is identical to the plume in (*a*) released at *t*_0_ = 0 at time *t*_0_, *t*_1_, …, *t*_4_, respectively. The superposition (sum) of discretized instantaneous sources released at time *t*_0_, *t*_1_, …, *t*_5_ constructs the isoconcentration profile of the plume, and makes it anisotropic in space. When Δ*t* tends to zero, the difference in the concentration distribution between instantaneous and continuous sources is highlighted at the bottom. The drawing does not take the wind speed gradient into account. See text for more details. (Online version in colour.)

If turbulent diffusion is not neglected in the vertical direction, and tephra is released instantaneously from a point source, the ash cloud would spread out isotropically in space, and look like a fuzzy, ball-shaped object (figure 1*a*). The plume would diffuse and move in the vertical direction due to falling at terminal velocity, and would diffuse and be advected in the horizontal directions due to wind as shown in figure 1*a*.

Whether volcanic ash is released instantaneously or continuously from a point source also affects the shape of the ash cloud and consequently the resultant tephra thickness distribution. For a continuous source with released particles having the same grain size, the source term can be decomposed to a sum of instantaneous sources released at each moment. The scenario can be better illustrated if time is discretized into *t*_0_ = 0, *t*_1_ = Δ*t*, *t*_2_ = 2Δ*t*,…, *t*_*c*_ = *c*Δ*t* as shown in figure 1*b*. As an example, at time *t*_5_, the plume released at moment *t*_1_ (ignoring sources released at other moments) would be identical to a plume originating at time *t*_0_ = 0 and observed at time *t*_4_. By a similar argument, the total plume concentration field with a continuous source at time *t*_5_ is the sum of concentration fields resulting from instantaneous releases at times *t*_0_, *t*_1_,…, *t*_5_. This argument shows that the iso-concentration surfaces for continuous releases are necessarily anisotropic and stretched in the direction of the wind and the fall velocity (figure 1*b*). Thus, because Ash3d assumes that tephra is released continuously from the source, turbulent diffusion is isotropic, and that wind speed gradient exists (not illustrated in figure 1, which would make the schematic drawing even more complicated), simulated results from Ash3d cannot be completely explained by the solution given in equation (2.7). However, the solution for the simplified semi-analytical case of equation (2.7) will be important for us in building an emulator.

## 3. Emulation design and testing

### (a) Simulated settings and data generation for training and validation

To reiterate, the challenges to constructing a robust GaSP emulator are (i) finding effective input variables to characterize wind conditions, and (ii) characterizing the net effect on the interaction between wind speed and tephra particles of various sizes (or fall velocities). As these are the major challenges in constructing emulators for volcanic ash models, we need to simplify the variability of other inputs as much as possible such that the results are not affected by other factors. In this way, we could better analyse the results, and provide more precise details and well-constrained insights on advantages and limitations of the emulators. We therefore ignore the variability of other inputs (e.g. turbulent diffusivity, eruption duration are fixed), and consider as input variables of interest: source height (*H*; km), total eruption volume (*V*; km^3^), tephra grain size distribution defined by mean and standard deviation *μ*_*gs*_ and *σ*_*gs*_ in the *ϕ* scale (equation (2.6)) and atmospheric conditions. The atmospheric conditions include the wind direction, which is assumed to be constant, and the wind speed, which varies with elevation but remains time-invariant. Wind speed in the vertical direction is set to zero. We use a Gaussian profile to describe the wind speed variation with height, which is defined by three parameters: the altitude at which the highest speed is reached (*μ*_*w*_; km) and the standard deviation (*σ*_*w*_; km) of the Gaussian profile of the wind speed, and the maximum wind speed (*w*_max_; m s^−1^). Due to the discretization of Ash3d (the height of each cell is 1 km for all simulations in this work; table 1), it should be noted that the column height of the simulations has discrete values. For example, simulations with source height from 10 to 11 km have their source cells all centred at 10.5 km.

**Table 1.** Range and value of initial conditions and parameters used to run Ash3d simulation for Settings One and Two.

variable	description	unit	range/value	reference
*V*	volume	km^3^	0.0001–1	Bursik & Sieh [86]
*H*	source height	km	6–35	
*μ*_gs_	mean grain size	*ϕ*	−5.5--4.5	Woods & Bursik [87]
*σ*_gs_	standard deviation of grain size	*ϕ*	0.6–3.8	Woods & Bursik [87]
*μ*_w_	mean of the Gaussian profile for wind speed	km	10–30	
*σ*_w_	standard deviation of the Gaussian wind speed profile	km	1–15	
*w*_max_	maximum wind speed	m s^−1^	0–50	
	tephra density	kg m^−3^	1000	
	cell size in *χ* and *ψ* directions	km	1 for Setting One	
			2 for Setting Two	
	cell size in the vertical direction	km	1	
	diffusion coefficient	m^2^ s^−1^	3000	
	eruption duration	h	1	

Construction of an emulator must sample from the entire space of physically plausible inputs. How probable is a particular subspace of inputs is a separate issue, important in a subsequent hazard calculation. The range adopted for the variables of interest listed above and value of the parameters that are fixed are given in table 1. Again, we stress here that the goal of the emulator is to estimate or approximate the output of a simulator, and the present work focuses on resolving two main challenges (stated above) in constructing emulators for volcanic ash transport models. The range and values of the ESPs and wind conditions chosen here (listed in table 1) would not affect any results or conclusions in this work as long as they are within reasonable ranges. Relatively wide ranges are chosen such that for the variables whose value is not fixed (variables whose variability is considered), we know that their variability would affect the simulated output. We use Latin hypercube sampling (LHS; [88–90]) to generate an experimental design of 10 000 points from the seven-dimensional input space (*V* × *H* × *μ*_gs_ × *σ*_gs_ × *μ*_w_ × *σ*_w_ × *w*_max_). We emphasize that, because the value of each variable is sampled independently, the specified column height, *H*, is not guaranteed to lie above *μ*_w_.

We construct a GaSP emulator for two special settings that illustrate our two main challenges. Setting One assumes that all erupted tephra particles are 0 *ϕ* in diameter, i.e. monodisperse. The goal for Setting One is to search for the appropriate transformed heuristic variables that better characterize impact of the wind speed profile on tephra thickness. Setting Two assumes that the grain size of released particles is described by a Gaussian distribution using the *ϕ* scale. In a way, Setting One can be regarded as a necessary intermediate step towards the emulator construction of Setting Two, the end goal of the present work.

For both settings, it is assumed that tephra particles are continuously released from a point source (a single cell in Ash3d) at a certain elevation (source height range: 6–35 km) for one hour, and that the wind blows southwards. We specify a location 30 km downwind from the source as the location of interest. We implement Ash3d for each Setting, with 10 000 simulations that sample the input space. We obtain 10 000 tephra thickness distributions for Setting One runs and 8082 for Setting Two runs. (Note in Setting Two, some simulations cannot be finished because only a limited amount of extremely fine particles is specified in such cases. The terminal velocity of these particles is low, and it takes much longer time than the specified simulation duration for them to deposit completely on the ground. These unfinished simulations do not affect any results and discussions listed below).

### (b) Emulator for Setting One: heuristic variable search

A simple test of directly emulating *h* and using *V*, *H*, *μ*_gs_, *σ*_gs_, *μ*_w_, *σ*_w_ and *w*_max_ as inputs failed (e.g. the conventional emulator construction. This test is not shown to avoid redundancy, but an example is given below demonstrating that we cannot construct emulators for Ash3d in the conventional way. That example is sufficient to prove that this simple test would fail). That is, the GaSP emulator approximated to *h* was dominated by uncorrelated noise. In this section, we propose physically motivated and transformed variables that will be used as inputs for the emulator. We also propose a scaling for the output.

#### (i) Using *h*/*V* as output

**Figure 2.**
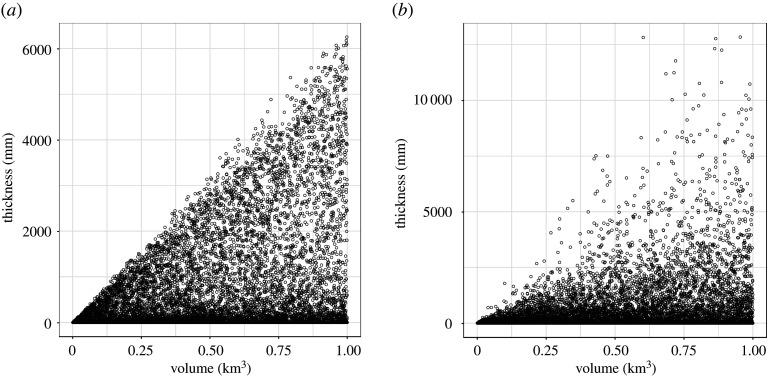
Simulated tephra thickness (mm) at the location of interest (30 km downwind from the vent) plotted against total eruption volume for Settings One (*a*) and Two (*b*).

Considering that directly emulating *h* would not work well, we recognize that tephra thickness scales with the total erupted volume—that is, doubling the erupted volume should result in a doubling of the thickness (approximately). Thus, we introduce a normalized thickness *h*_*V*_ = *h*/*V* (figure 2) as output for both Settings One and Two.

With this output transformation, we will not use *V* as an input for constructing emulators. Note that we could make *h*_*V*_ unitless by dividing by *V*^1/3^ instead of *V*, however, this is not done because we want to keep the transformation as simple as possible. Also, we have attempted to use *h* as output variable and/or include *V* as input variable to the emulator for all experiments implemented in this work, and the corresponding results do not improve. They are not shown here to avoid redundancy.

#### (ii) Using advected distance to characterize the wind conditions

We propose three transformed input variables that are functions of the advected distances to characterize the wind speed. Here we examine what the variables should be and what features they should reflect.

Although in Setting One only one grain size is considered, the proposed variables should function well and also be compatible with Setting Two. As pointed out earlier, one major challenge in constructing emulators for Ash3d is that tephra particles of various sizes react differently to the same wind condition, and we do not know what the particle sizes that could affect the simulated thickness at a location of interest are. This is an issue for Setting Two. If this problem can be solved, it can be expected that the solution might require us to subdivide the problem based on grain sizes, and analyse them separately (which is the case as will be shown later). Therefore, we argue that the transformed input variables should be functions of both wind speed and particle size.

Advected distance denotes the distance a particle within the plume travels horizontally due to wind advection, and fits well with the requirement listed above. Therefore, we use combinations and transformations of advected distances, rather than wind speed, at elevations below *H*, to characterize the wind conditions. This would also avoid including non-effective information, namely wind speed well above the source, into the emulator construction. Note that the source height *H* might lie below or above the altitude with maximum wind speed, which, we will find, complicates the emulator construction and requires compensation.

We examine features of the wind speed and advected distance profiles to provide intuition on how to determine good candidates for transformed input variables. First, the integral of wind speed below the source height plays an important role in determining tephra dispersal, which is also true for the advected distance. This is intuitive and indicated in equation (2.7) in discrete form. That marks one transformed input variable to characterize the wind speed. Note that the integral is replaced by the summation of advected distance at elevation levels below *H* due to the discretization in Ash3d.

Next, consider the relatively complicated case where the source height (*H*) is above the mean in height of the Gaussian profile (*μ*_w_; the altitude of the maximum wind speed). The wind speed first increases with elevation until *μ*_w_, and then starts to decrease. The advected distance might follow a similar fashion, and has a peak (along the vertical direction) below *H*. It should be noted that this peak might not be at *μ*_w_, and that the advected distance profile is not symmetric with respect to this peak, as the terminal velocity varies with elevation (see the calculation of advected distance in §2c). From our experiments, we find that advected distance profiles have various shapes, and cannot be uniformly represented by a single functional form such as a second or third order polynomial, or squared exponential function. As the advected distance profile is not symmetric with respect to the elevation that has the maximum advected distance, shapes of the advected distance profile above and below the maximum advected distance elevation are different, and thus need to be characterized separately. That is to say (at least) two more features or variables (features above and below the height with the maximum advected distance), in addition to the total advected distance, are needed to characterize the advected distance profile.

In other situations where the the advected distance increases with elevation monotonically, a peak in the advected distance profile does not exist. In such a case, we cannot characterize the advected distance profile below and above the peak. To avoid this problem, we select one transformed input variable to characterize the overall variability of the advected distance profile, in addition to the integral (sum) of advected distances below *H*, and another to characterize the profile with a focus on elevations closer to the source height. We expect these two features to be able to characterize the shape of the wind speed and advected distance profiles, and from a physical perspective, reflect the overall wind shear below *H* and wind speed closer to the source height, respectively.

Based on the above arguments, we propose three variables to characterize the wind speed profile in the sections below.

#### (iii) Total advected distance

We examine as a variable the total advected distance of a particle with grain size *ϕ*(*j*)

3.1 $$l_{j}=\sqrt{{\left(\sum_{i=1}^{n}\delta \chi_{i,j}\right)}^{2}+{\left(\sum_{i=1}^{n}\delta \psi_{i,j}\right)}^{2}}.$$

Of course, in Setting One, we only have particles of size *ϕ*(0). Because we assume a northerly wind, hence $\sum \delta \chi_{i}=0$, the advected distance can be simplified as $\sum_{i=1}^{n}\delta \psi_{i,j},$ assuming that the positive *χ* and *ψ* directions are to the east and south, respectively. This variable denotes the integral of the advected distance below *H*. Physically, the total advected distance represents the horizontal distance a particle travels from being released to reach the ground due to wind advection. The proposition of total advected distance is indicated in equation (2.7).

**Figure 3.**
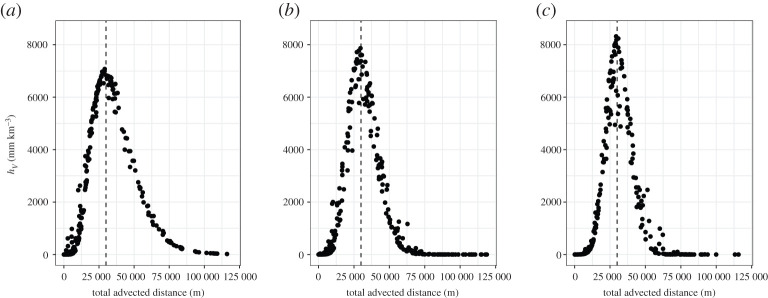
Transformed tephra thickness *h*_*V*_ = *h*/*V* plotted against total advected distance *l*_*j*_ for Setting One simulations with source height being 10.5 (*a*), 20.5 (*b*) and 30.5 (*c*) km. The vertical dashed line corresponds to the total advected distance of 30 km.

We select and group simulations with source height of 10.5, 20.5 and 30.5 km into three subsets (due to the discretization of Ash3d as mentioned earlier), and examine how *h*_*V*_ varies with total advected distance in figure 3 for each subset. Note that for Setting One, the goal is to find effective variables to characterize the wind conditions. Ignoring the variability of the source height in the following Setting One experiments avoids its impact on the output, and the output *h*_*V*_ is only affected by the wind conditions in this way.

There is a clear trend showing *h*_*V*_ varying with the total advected distance. When advected distance equals 30 km, the location of interest in our study, the deposit thickness is maximal. The thickness gradually decreases as the advected distance increases beyond 30 km.

#### (iv) Weighted advected distance and sum of squared advected distance

The patterns shown in figure 3 display two features that cannot be explained by the simplified model (equation (2.7)) or the total advected distance. First, note that *h*_*V*_ is not symmetric with respect to the 30 km maximum. Second, with greater column height *H*, the variability in *h*_*V*_ increases and cannot be explained by the total advected distance alone.

As mentioned earlier, in addition to total advected distance, we need two other variables to characterize the advected distance profile. One focuses on the part that is at elevations closer to *H*, and the other on its overall variability. The two transformed variables we propose are named weighted advected distance (*λ*_*j*_) and the sum of the squared advected distance (*τ*_*j*_). Their definitions are given below.

The weighted advected distance is defined as:

3.2 $$\lambda_{j}=\sum_{i=1}^{n}\sqrt{\left(\delta \chi_{i,j}^{2}+\delta \psi_{i,j}^{2}\right)}w_{i,j},$$

where *w*_*i*,*j*_ is calculated as:

3.3 $$\begin{array}{rl} w_{i,j} & =\frac{1/T_{i,j}}{\sum_{i=1}^{n}(1/T_{i,j})}\\ \;\text{and}\;T_{i,j} & =\sum_{k=i}^{n}t_{k,j}, \end{array}\}$$

where *T*_*i*,*j*_ denotes the time for a particle with grain size *ϕ*(*j*) to fall from the source height to elevation level *H*_*i*_. The weight *w*_*i*,*j*_ is constructed to give greater weight to advected distance at elevations closer to *H*. This variable is defined to characterize features of the advected distance profile with a focus on elevations closer to the source height.

The sum of the squared advected distance (*τ*_*j*_) is defined as:

3.4 $$\tau_{j}=\sqrt{\sum_{i=1}^{n}(\delta \chi_{i,j}^{2}+\delta \psi_{i,j}^{2})}.$$

This variable is used to characterize the overall variability of the advected distance profile, namely the overall vertical wind speed gradient. Note that the wind speed gradient or the advected distance gradient along the vertical direction has different values at different elevations, and the proposed *τ*_*j*_ gives a general characterization of the gradient below *H*. We note that weighted advected distance and sum of squared advected distance are proposed heuristically, but they are motivated by the interaction between shape of the wind speed profile, source height and released particle size.

#### (v) Testing and comparison with conventional emulator construction

We now test the three proposed transformed input variables *l*_*j*_, *λ*_*j*_ and *τ*_*j*_ as inputs for the emulator for Setting One. We focus on Setting One simulations with source height of 10.5, 20.5 and 30.5 km. In this way, the results are not affected by the source height. We compare results derived from the original inputs and transformed inputs in the following five combinations:

(1) The original variables (*μ*_w_, *σ*_w_ and *w*_max_);
(2) Total advected distance (*l*_*j*_) alone;
(3) Total and weighted advected distances (*l*_*j*_ and *λ*_*j*_);
(4) Total advected distance (*l*_*j*_) and sum of squared advected distance (*τ*_*j*_);
(5) Total and weighted advected distances (*l*_*j*_ and *λ*_*j*_) and sum of squared advected distance (*τ*_*j*_).

Note that Combination 1 represents the conventional emulator construction, that is, using the parameters that define the wind speed profile as inputs to train the emulator. Combinations 2–4 take one or two of the three proposed variables as inputs to train the emulator, they are tested here to see whether using three variables to characterize the wind speed profile (advected distance profile) is necessary. Combination 5 represents the proposed emulator construction.

For each input scenario, 80% of the simulation outputs are selected and used to train a GaSP emulator, and the remaining 20% are used to test the emulator. This process is done five times with no redraws of testing samples (fivefold validation), such that all samples are used for testing once. All validation results presented below, including those of Settings One and Two, are from such a fivefold validation procedure. We use four measures to evaluate the results. They are (i) empirical frequency coverage (EFC) of a function by credible intervals from the emulator, the percentage of simulated *h*_*V*_ that is within the 95% emulated confidence interval; (ii) root-mean-square predictive error (RMSPE) between the simulated *h*_*V*_ and emulated mean of *h*_*V*_; (iii) averaged emulated standard deviation of *h*_*V*_ (${\bar{\text{std}}}_{\text{emu}}$); and (iv) RMSPE/RMSPE_base_, where RMSPE_base_ denotes the standard deviation of the simulated *h*_*V*_ within each subset. The last measure has a range of 0–1, and indicates the fraction of variability that cannot be explained by variability in the emulations, and does not scale with the output *h*_*V*_. Good validation results correspond to greater values for EFC (range: 0–100%).

**Figure 4.**
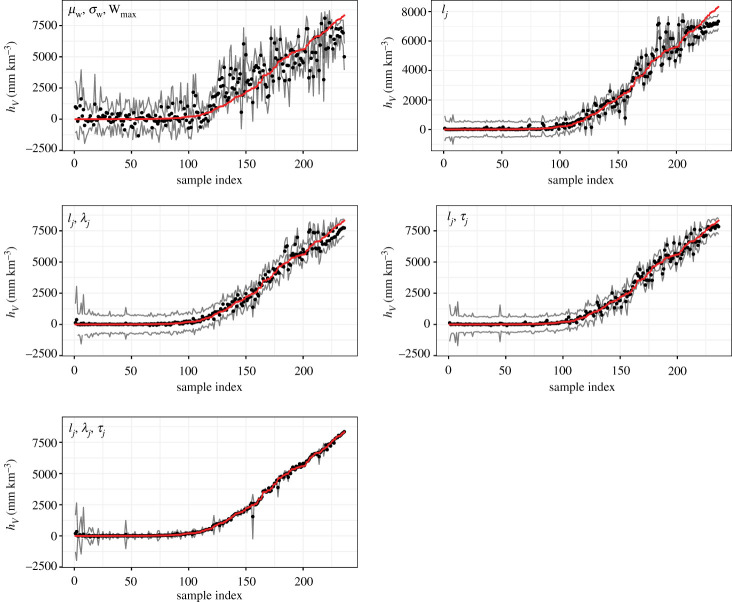
Setting One fivefold validation results for the subset with source height of 30.5 km using different combinations of input variables to train the emulator. The input variables for the five emulators are marked in the upper-left corner of each plot (see text for more details). The fivefold validation is done by taking one fifth of the total samples out for testing and the rest for training five times, and samples are drawn for testing only once (no redraw of samples for testing). Every sample within each subset has been used for testing once. After the validation is done, an index is given to each testing point based on the (true) value of the simulated *h*_*V*_ in ascending order. The emulated mean (black point), emulated 95% confidence interval (grey lines) and the corresponding simulated value of *h*_*V*_ (red line) are shown and plotted against the index. (Online version in colour.)

Validation results for the three subsets are summarized in table 2 and in figure 4 for the subset with source height of 30.5 km. In most cases, more than 90% of the testing values of *h*_*V*_ are within the 95% emulated confidence interval as suggested by the EFC values. Combination 5 with the proposed transformed input variables usually outperforms the other four proposals by a factor of 2 or more, suggesting that the proposed emulator outperforms the conventional emulator (table 2), and three variables are needed to characterize the wind speed or advected distance profile (figure 4).

**Table 2.** Summary of Setting One validation results for subsets with source height of 10.5, 20.5 and 30.5 km. The subset size and range of *h*_*V*_ for each subset are given. EFC, RMSPE, ${\bar{\text{std}}}_{\text{emu}}$ and RMSPE/RMSPE_base_ are used to evaluate the performance of the emulator. The five columns correspond to results from using different combinations of input variables to train the emulator. See text for more details.

column height: 10.5 km (subset size: 346; range of $h_{V}:0-7075.11$)					
input combination	1	2	3	4	5
EFC(%)	93.06	95.09	95.38	88.44	95.38
RMSPE	584.71	1330.65	58.56	175.12	57.91
${\bar{\text{std}}}_{\text{emu}}$	519.15	1073.92	62.29	150.06	62.71
RMSPE/RMSPE_base_	0.241	0.549	0.024	0.072	0.024

column height: 20.5 km (subset size: 307: range of $h_{V}:0-7866.89$)					
input combination	1	2	3	4	5
EFC(%)	93.16	95.11	95.11	91.21	90.55
RMSPE	956.67	405.52	248.95	323.73	139.34
${\bar{\text{std}}}_{\text{emu}}$	950.27	409.18	274.77	297.01	124.33
RMSPE/RMSPE_base_	0.394	0.167	0.102	0.133	0.057

column height: 30.5 km (subset size: 236: range of $h_{V}:0-8318.63$)					
input combination	1	2	3	4	5
EFC(%)	92.37	94.49	93.64	91.53	91.95
RMSPE	1080.77	487.47	343.65	296.23	88.12
${\bar{\text{std}}}_{\text{emu}}$	1065.04	506.20	381.63	322.64	91.79
RMSPE/RMSPE_base_	0.405	0.183	0.130	0.111	0.040

It is of note that combination 3 performs closest to combination 5, especially for the lowest source height of 10.5 km. This is because within this subset, *μ*_w_ is always above 10.5 km (*H*), and the advected distance always increases with elevation till the height of the source. Performance of the fifth combination is most clearly visible for the highest source *H* = 30.5 km. We note here that it is apparent that Combination 1, the conventional emulator construction, is unable to perform as well as the proposed emulator construction (figure 4) in the simplest scenario considered. Its performance would only become worse when more complicated scenarios (i.e. Setting Two) are considered. Thus, it is not necessary to compare the conventional emulator construction with the proposed one in the following tests.

### (c) Emulator for Setting Two

#### (i) Subsetting based on the dominant grain size

Setting Two introduces two additional dimensions into the emulator construction, the mean and standard deviation of the grain size distribution, *μ*_gs_ and *σ*_gs_. It is in incorporating grains of variable sizes that the challenges mentioned in the previous section become fully apparent. We propose to identify the grain size that has the greatest contribution to *h*_*V*_ for a given simulation, and group the simulations based on this grain size. We refer to this as the dominant grain size. The dominant grain size can be pre-calculated based on the simplified model of equation (2.7). That is, we apply equation (2.7) to each grain size, and the one that has the greatest mass per unit area, i.e. the main mode, is considered the dominant grain size. We then calculate *l*_*j*_, *λ*_*j*_ and *τ*_*j*_ based on the dominant grain size within each subset, and the training (parameter fitting) is done separately.

The major advantage of subsetting based on the dominant grain size is that the subsetting takes into account the interplay of wind speed, source height and particle size. In this way, the most effective (in terms of determining *h* or *h*_*V*_) grain size for samples within each subset is known. Therefore, we expect this measure to have the ability to resolve the second challenge of building emulators for volcanic ash transport models stated above.

#### (ii) Testing

We test the performance of the proposed measures, i.e. transformation of input and output variables and subsetting based on the dominant grain size, in constructing emulators for Setting Two in this section. The testing is done for each subset separately.

The dominant grain size of the samples typically ranges from −6 to 4 *ϕ* in our Setting Two experiments. As subsets with −6 and 4 *ϕ* contain only one and eight samples, respectively, we will ignore these and focus on the remaining subsets. Thus, the transformed input space for Setting Two includes source height (*H*), *μ*_gs_, *σ*_gs_, *l*_*j*_, *λ*_*j*_ and *τ*_*j*_ calculated based on the dominant grain size. The variability in all specified initial conditions and parameter values (variables listed in table 1 with range given), including the source height, is taken into account in the following experiments.

**Figure 5.**
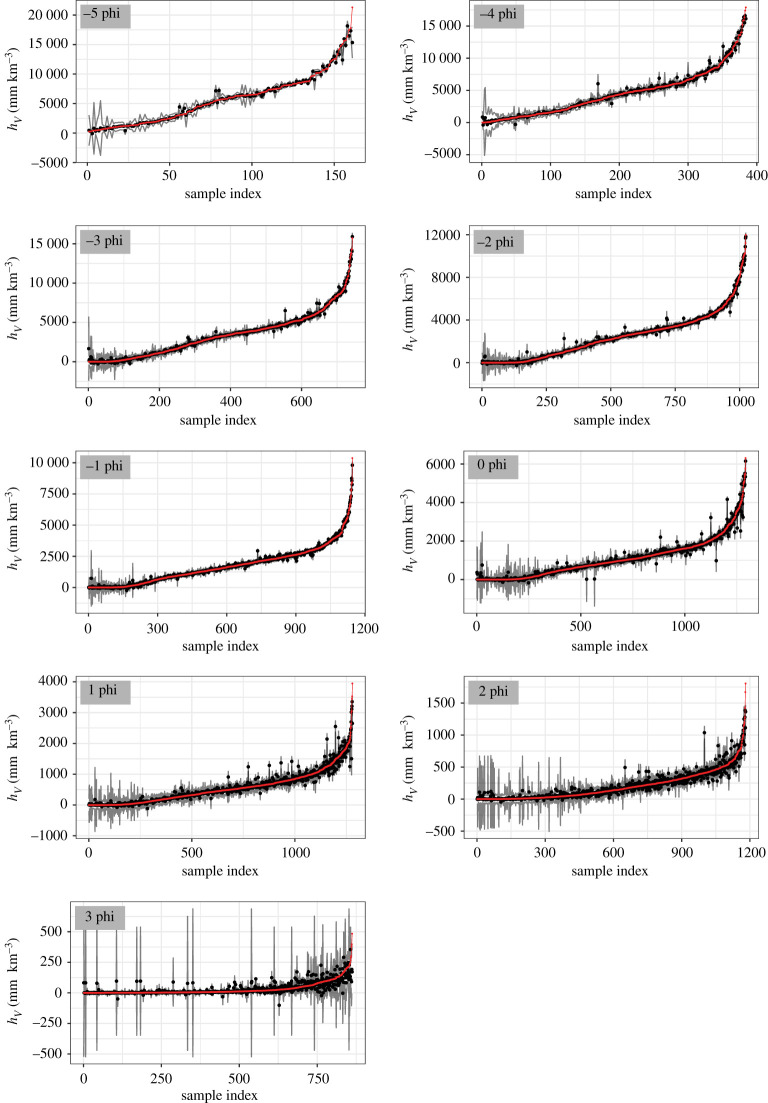
Setting Two fivefold validation results for subsets with different dominant grain sizes (marked in the upper-left corner of each plot). The fivefold validation is done by taking one fifth of the total samples out for testing and the rest for training five times, and samples are drawn for testing only once (no redraw of samples for testing). Every sample within each subset has been used for testing once. After the validation is done, an index is given to each testing point based on the (true) value of the simulated *h*_*V*_ in ascending order. The emulated mean (black point), emulated 95% confidence interval (grey lines) and the corresponding simulated value of *h*_*V*_ (red line) are shown and plotted against the index. Note that the scale of *y*-axis is different for each plot. (Online version in colour.)

**Figure 6.**
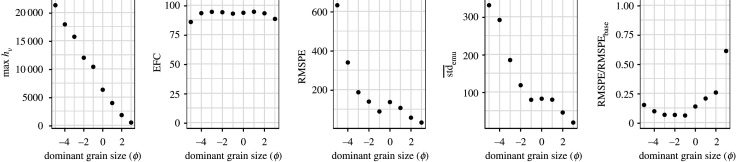
Maximum *h*_*V*_, EFC, RMSPE, ${\bar{\text{std}}}_{\text{emu}}$ and RMSPE/RMSPE_base_ for each subset from the fivefold validation test for Setting Two. In each plot, the *x*-axis denotes the dominant grain size of the subset in *ϕ* unit. The figures are plotted based on table 3.

We apply fivefold validation to each subset (based on the dominant grain size), and the results are shown in figure 5. Setting One experiments suggest that emulating the original output with original input variables of Ash3d, namely the conventional emulator construction, performs poorly, and we will not explain that case in Setting Two. We also provide an evaluation based on EFC, RMSPE, ${\bar{\text{std}}}_{\text{emu}}$ and RMSPE/RMSPE_base_ in table 3 and figure 6.

**Table 3.** Summary of Setting Two validation results for subsets with dominant grain size ranging from −5 to 3 *ϕ* and subsets with dominant grain size of 3 and 2 *ϕ* and negligible total advected distance. The latter is done to show that the performance of the emulator improves for simulations with finer dominant grain size that are not affected by strong wind. The subset size and range of *h*_*V*_ (unit: mm km^−3^) for each subset are given. EFC, RMSPE, ${\bar{\text{std}}}_{\text{emu}}$ and RMSPE/RMSPE_base_ are used to evaluate the performance of the emulator.

dominant grain size (phi) and other					
criteria for subsetting (subset size)	max (*h*_*V*_)	EFC	RMSPE	${\bar{\text{std}}}_{\text{emu}}$	RMSPE/RMSPE_base_
−5 (161)	21297.64	85.71	631.04	330.71	0.150
−4 (384)	17902.70	93.23	338.74	291.50	0.096
−3 (743)	15708.14	94.35	186.44	184.77	0.066
−2 (1024)	11980.68	94.04	138.84	117.84	0.065
−1 (1145)	10374.15	92.84	88.27	79.06	0.060
0 (1292)	6307.35	93.58	136.46	81.97	0.137
1 (1279)	3947.02	94.45	106.50	79.41	0.205
2 (1181)	1806.54	93.14	56.80	45.24	0.256
3 (862)	483.97	88.34	31.84	18.52	0.610
2 & total advected distance <0.01(47)^a^	46.26	91.49	1.97	1.54	0.204
3 & total advected distance <1(239)^a^	106.60	93.29	1.90	1.38	0.166

^a^Input variables: source height, mean and standard deviation of grain size distribution.

The maximum *h*_*V*_ for each subset ranges from 21 297 to 483, and decreases with the dominant grain size. The EFC values suggest that most subsets have more than 90% of the simulated *h*_*V*_ within the 95% emulated confidence interval. The exceptions are the subsets with the coarsest and finest dominant grain sizes, which have slightly lower EFC (85.71 and 88.34%). Both RMSPE and ${\bar{\text{std}}}_{\text{emu}}$ generally decrease with the dominant grain size, ranging from 631 to 31 and 330 to 18, respectively. These two measures scale with the range of *h*_*V*_ and cannot be directly used for comparison between subsets.

The variable scale of *h*_*V*_ for the different subsets suggests that we should re-examine the ratio RMSPE/RMSPE_base_ to evaluate the Setting Two emulator construction. RMSPE/RMSPE_base_ is 0.150 for the subset with dominant grain size of −5 *ϕ*. This value is relatively high because the subset only has 161 simulations. The RMSPE/RMSPE_base_ value is below 0.100 for subsets with dominant grain size ranging from −4 to −1 *ϕ*. For the other subsets (dominant grain size range: 0 to 3 *ϕ*), RMSPE/RMSPE_base_ increases with the decrease in dominant grain size, ranging from 0.137 to 0.610.

The comparison suggests that our emulator fits to the transformed input variables well in most cases, especially for subsets characterized by coarser dominant grain size. Less accurate results with increased uncertainty are obtained when the emulator is applied to subsets with finer dominant grain size. By exploring the relationship between the inputs and output from the simulation data (i.e. training samples), we find that this is related to three main factors: (i) simulations could have the same dominant grain size under two physically distinct scenarios: with or without wind; (ii) dominant grain size calculated from the semi-analytical solution (equation (2.7)) and determined from Ash3d simulation could be different even with the same initial conditions; and (iii) we neglect the wind speed above the source height in calculating values of *l*_*j*_, *λ*_*j*_ and *τ*_*j*_. These sources of variability are more likely to occur for subsets characterized by finer dominant grain size. They will be discussed in more detail in the following section.

## 4. Discussion

Our work has demonstrated and validated our emulator based on transformed input and output variables and subsetting the training data by the dominant grain size.

### (a) Novelties

Our work provides a new perspective on the emulator construction for geophysical simulators. Its core ideas can be summarized as (i) finding and using input and output variables that better capture the dominant physics of the system, i.e. finding the appropriate input and output spaces for training the emulator; and (ii) partitioning the input space based on knowledge of the simulated process, and training the emulator separately by subsets.

Our approach deals with a common problem for the design of machine learning techniques: how one should merge machine learning techniques with knowledge on the process being analysed. In our case, the simulated physical process is controlled by the advection–diffusion equation solved by the simulator Ash3d. We focus on the relatively simple process, advection, to transform the input space of the emulator. Wind advection blows the plume or particles within the plume in a simple and linear way. The effect of wind advection must be denoted by either wind velocity- or distance-related variables which are functions of wind speed. The advection is also affected by the time particles spend in the air, and therefore the proposed variables should be a function of time and grain size. This narrows down the potential variables to be combinations or transformations of the advected distance.

Proposed total advected distance (*l*_*j*_) is indicated and implied by the simplified semi-analytical solution of tephra dispersal (equation (2.7)). In addition, we point out another two features of the wind speed or advected distance profile that are not reflected in the total advected distance, and propose two variables (*λ*_*j*_, and *τ*_*j*_) to characterize them accordingly. The proposition of their utility is heuristic, but comparison of the validation results in Setting One suggests that at least the emulator outperforms a standard emulator, namely using untransformed, raw input variables of the simulator to train the emulator.

Using these variables as inputs to characterize the wind conditions can be regarded as a kernel trick (e.g. [1,91]). The kernel trick does not attempt to project the input space into higher dimensions, but aims at finding the physically sound and effective variables to represent the input space. Emulating *h*_*V*_ instead of *h* follows a similar idea.

The second core idea deals with Setting Two. With the help of our prior knowledge of the physics of tephra transport (i.e. equation (2.7)), we are able to find out what grain sizes are active or not in determining the value of *h* or *h*_*V*_ at the location of interest. We pick the grain size that has the greatest contribution to *h*_*V*_ or *h* as the dominant grain size, and use it as the criterion for subsetting. Simulations within each subset are dominated by the same type of wind-particle size interaction, and are ‘closer’ to each other in the transformed input space.

Our work represents an effective emulator construction for simulators that solve the advection–diffusion equation with a continuous point source, constant (turbulent) diffusion coefficient and relatively complex velocity field. The methodology could possibly benefit the emulator construction for other simulators that solve the same or similar governing equations. The work also sets an example on how to improve the performance of the GaSP emulators by using some physical knowledge of the simulator.

### (b) Simplifications in our emulator construction

#### (i) Simplifications in running Ash3d and their justifications

*Point source in space*. We assume that particles are released from a point source rather than a line source in all simulations in the present work. This simplification is valid for two reasons: (i) most volcanic ash is released from the top of the eruptive column; and (ii) similar to the discretization of a continuous source in time, an eruptive column could also be discretized as point sources in space. Assuming a point source in all simulations could help us focus on major concerns of the present work.

*Constant wind direction*. Constant north wind is assumed for all simulations, and the effect of cross wind is neglected. Again, this simplification is proposed such that we could focus on the main concerns of the present work. Nonetheless, for emulating scenarios with cross wind, one could simply decompose the wind speed into two perpendicular vectors, calculate values of *l*_*j*_, *λ*_*j*_ and *τ*_*j*_ for the two directions separately and use them to train the emulator. This would indeed require more simulator runs for collecting training data, but is unavoidable given the increased complexity of the simulated process.

*Other fixed initial conditions*. We keep some initial conditions and parameter values fixed in both Settings One and Two simulations, including turbulent diffusivity, eruption duration and tephra density. These variables affect the simulated process in a relatively straightforward way: a change in the value of these variables would definitely lead to a change (possibly very small) in the output. To take their variability into account, one simply needs to run more simulations to deal with the increased dimensionality of the input space or implement sensitivity analysis to examine their impact on the model output.

### (c) Sources of uncertainty

As validation results for Setting One have low uncertainty, our discussion here focuses on Setting Two. Its validation results suggest greater uncertainty for subsets with finer dominant grain size. In this section, we point out sources of uncertainty, and list proposed measures (and comments) to avoid or reduce their impact on the performance of the emulator.

#### (i) Same dominant grain size for simulations with and without wind

In the absence of wind, the source height and grain size distribution characterize the simulated process, and determine the dominant grain size. Simulations with and without the presence of wind could have the same dominant grain size. For the latter, however, it is not necessary to include advected distance-related variables as inputs to train the emulator.

**Figure 7.**
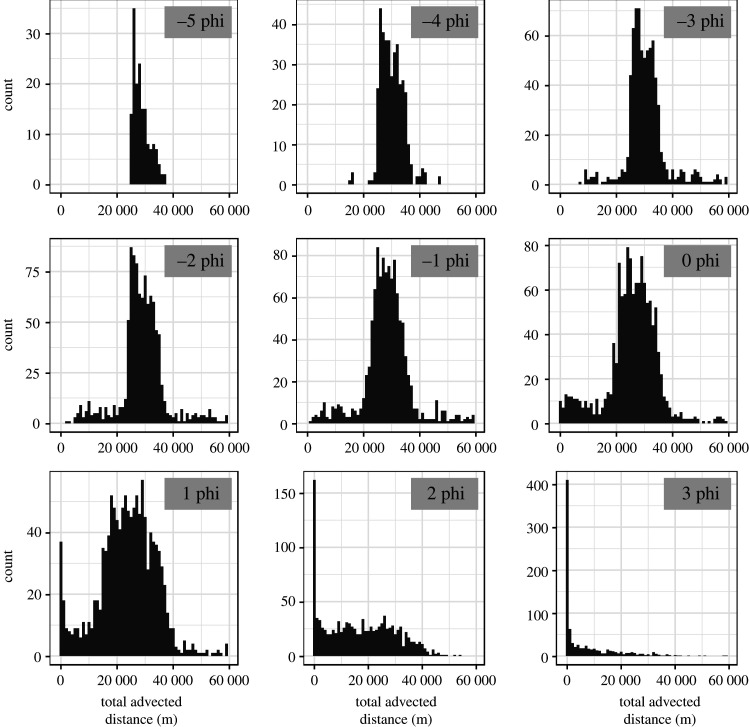
Distribution of total advected distance for Setting Two simulations within each subset.

Grouping those simulations that are affected by and not affected by the wind into a single large subset tends to occur more frequently for subsets with finer dominant grain size. This is because finer particles have low terminal velocity, and even in the absence of wind, could be transported to locations that are relatively far from the source due to turbulent diffusion, and become the dominant grain size. This suggests that for subsets with finer dominant grain size, the distribution of total advected distance, an indicator of the overall wind speed, should be bimodal (one mode close to zero, dominated by turbulent diffusion, and the other being much greater, dominated by wind advection) or heavy-tailed. The histogram of total advected distance is plotted for each subset (figure 7), which confirms this argument: bimodal and heavy-tailed distributions occur for subsets with dominant grain sizes of 1–3 *ϕ*.

This source of uncertainty could be easily reduced by further grouping the subsets with finer dominant grain size based on the total advected distance, and train them, and make the prediction separately. To test this, simulations with a dominant grain size of 2 and 3 *ϕ* and low total advected distance are selected. We use source height, mean and standard deviation of grain size (without using advected distance-related variables) as inputs to train and test the emulator with these data through fivefold validation. The results suggest that the performance of the emulator improves greatly (table 3). This proposition only requires one or a few more subsets with fewer input variables, and thus does not increase the complexity of the current emulator. Having more training samples with finer dominant grain size also alleviates this issue.

#### (ii) Difference between semi-analytical solution and Ash3d simulation

Due to the difference between the semi-analytical solution and what Ash3d simulates, it is possible that the dominant grain size calculated from the two is different given the same initial conditions. As finer tephra tends to spend more time in the atmosphere, the difference between the semi-analytical solution and results from Ash3d simulation is relatively greater. This increases the likelihood of incorrect calculation of the dominant grain size.

To reduce this source of uncertainty, one could determine the dominant grain size by first constructing Setting One emulators. One could estimate the contribution to *h*_*V*_ from each grain size for Setting Two based on the Setting One emulators, and determine the dominant grain size, and thus avoid the use of the semi-analytical solution. It should be noted that this does not require a lot of Setting One simulations, as we only need to focus on Setting One simulations (which are also a lot faster compared with Setting Two simulations) with finer grain sizes.

#### (iii) Neglecting wind speed above the source height

The calculation of *l*_*j*_, *λ*_*j*_ and *τ*_*j*_ neglects the wind speed above the source height. In the presence of turbulent diffusion in the vertical direction, a certain portion of the tephra particles would be transported upwards from the source due to the concentration gradient. Wind speed above the source thus plays a role in determining the value of *h*_*V*_. Ignoring it introduces added epistemic uncertainty to our emulator construction. Finer particles sent above the source are more affected by the wind there because of their lower terminal velocity. This also explains why greater uncertainty occurs for subsets with finer dominant grain size. How the output *h*_*V*_ is affected by this source of uncertainty requires further investigation. Finding a way to avoid or reduce this source of uncertainty marks one main challenge for the refinement of the current emulator construction.

### (d) Implication

It is common to use LHS to generate sample points in the input space for the emulator. Our work has shown that greater uncertainty tends to occur for simulations characterized by finer dominant grain size. We could improve the current emulator by having fewer training samples with coarser dominant grain size, and more samples with finer dominant grain size, as the dominant grain size can be determined by the semi-analytical solution beforehand. Alternatively, we could also generate samples from the transformed input space for the emulator, instead of the raw, untransformed input space of Ash3d, for the emulator construction. Once these samples are generated, they could be transformed back to the input space of Ash3d, and used as initial conditions for simulation. This ensures that the training points would be evenly distributed in the input space of the emulator.

## 5. Conclusion

We have presented a GaSP emulator construction strategy that can be used to predict simulated tephra thickness from the numerical model Ash3d. Our work focuses on addressing two key concerns, namely, finding the effective input variables for the emulator to denote the wind conditions, and dealing with the complex interaction between wind conditions and tephra particles of different grain sizes. Its main assumptions include: (i) volcanic ash with identical density is released from a point source continuously; (ii) isotropic turbulent diffusion; (iii) wind direction does not vary with elevation, and wind speed is non-zero only in the longitude and latitude directions; and (iv) wind speed can be described by a Gaussian profile in the vertical direction.

Our work acknowledges that knowledge of the physics of tephra transport benefits emulator construction. Instead of just focusing on the raw inputs and output of the simulator, we propose to transform them for the emulator, and subset the training data based on the dominant grain size to improve the emulator. From a machine learning perspective, the two novelties can be phrased as: based on our prior, physical knowledge on the analysed process, we (i) find and adopt the appropriate input and output variables for the emulator by transformation of the original input variables of the simulator; and (ii) determine the dominant factor that affects the output, namely the dominant grain size, and group the training data based on it, and train them separately by subsets. Our emulators outperform normal emulators which simply use the raw input and output variables of the simulator for training and making prediction.

Sources of uncertainty for our design derive from the subsetting rule, differences between the semi-analytical solution and Ash3d simulation, and neglecting the effect of wind speed above the source height. The first two sources of uncertainty can be reduced or avoided by further subsetting based on whether a simulation was affected by wind or not, collecting more training data preferentially, and determining the dominant grain size based on Setting One emulators rather than the semi-analytical solution. The third source of uncertainty requires further investigation (e.g. sensitivity analysis) and marks a new challenge for further refinement of the current emulator construction.

Our work represents a general physically motivated emulator construction methodology for numerical models that solve the advection–diffusion equation with a relatively complex velocity field. It could be potentially applied to probabilistic hazard analysis and efficient inversion of volcanic eruptions. We hope that core ideas of our emulator construction and discussions of them would benefit future studies with similar goals and inspire the fusion of other machine learning techniques with complex numerical models.

## Data accessibility

The data used in the manuscript can be found at: https://vhub.org/resources/4632.

## Appendix A

**Table:**

	notations defined in §2a
***x***:	input for the simulator (a point in the input space)
**y**^*M*^(***x***):	simulator output
***h***(***x***):	basis functions
**β**:	vector of coefficients for ***h***(***x***)
*Z*(***x***):	zero-mean autocorrelated Gaussian process
*R*_*ij*_:	correlation between *z*(***x***_*i*_) and *z*(***x***_*j*_)
***x****	untested point in the input space
**R**:	correlation matrix for *Z*(***x***)
$\hat{y}(x^{*})$:	estimated mean of the emulator
*s*^2^(***x****):	variance of the emulator estimate

	notations defined in §2b
*q*:	tephra concentration
**u**:	3-D wind speed vector
*v*:	terminal velocity
*K*:	turbulent diffusivity
*Q*:	source term

	notations defined in §2c
*m*_*j*_(*χ*, *ψ*):	mass per unit area for grain size *ϕ*(*j*) at location (*χ*, *ψ*)
*M*_*j*_:	total mass for grain size *ϕ*(*j*)
*δχ*_*i*,*j*_, *δψ*_*i*,*j*_:	distance a tephra particle with grain size *ϕ*(*j*) travels within the *i*th horizontal layer in the *χ* and *ψ* directions due to wind advection
*t*_*i*,*j*_:	falling time within the *i*th horizontal layer (Δ*H*_*i*_) for particles with grain size *ϕ*(*j*)
*T*_0,*j*_:	falling time from source height *H* to the ground for particles with grain size *ϕ*(*j*)
Δ*H*_*i*_:	height of the *i*th horizontal layer
*v*_*i*,*j*_:	terminal velocity of particles with grain size *ϕ*(*j*) within the *i*th horizontal layer.
*h*:	tephra thickness
*h*_*V*_:	tephra thickness divided by total volume (*V*)
